# The Pathogenesis of Cholelithiasis in the Light of Latest Investigations

**Published:** 1899-06

**Authors:** Francis E. Fronczak

**Affiliations:** Assistant surgeon, Sisters of Charity Hospital; physician to the Erie County Penitentiary; visiting physician and surgeon to the Felician Institute, at Cheektowaga, N. Y., etc.


### Translation.

### THE PATHOGENESIS OF CHOLELITHIASIS IN THE LIGHT OF LATEST INVESTIGATIONS.

REVIEWED BY DR. STANISLAUS PECHKRANC, in *Medycyna*, Warsaw. Tom. R. XXVII., Nos. 6 and 7. Translated from the Polish, with Annotations.

By FRANCIS E. FRONCZAK, A. M., M. D.,
Assistant surgeon, Sisters of Charity Hospital; physician to the Erie County Penitentiary; visiting physician and surgeon to the Felician Institute, at Cheektowaga, N. Y., etc.

AS YEARS AGO, so also now there are two different views on the pathogenesis of cholelithiasis. Some believe it to be a dyscrasia, others seem to think that local changes in the biliary ducts are the cause of the condition. The first theory seemed to have enjoyed the greater confidence and hence was more believed than the latter. And yet, time and again, scientific men of reputation raised their voices against it. Forty years ago the great Frederichs denied the existence of special cholelithiatic diathesis, and preached the doctrine that these growths are the results of local disturbances. But not only Frederichs had this view on the subject, for Luton, Niemeyer, Barth, Besnier and others spread the same teachings, by word of mouth and by many writings.

The dyscratic theory, however, found a most ardent supporter in the person of Bouchard, who brought this theory to its highest possible standard. According to Bouchard, cholelithiasis, similar to gout, rheumatism, obesity and many others, is only an expression of a hereditary or acquired condition, due to the slow assimilation of food and deranged nutrition. This deranged assimilation and nonnutrition have as a result the abnormal collection of cholesterin in the bile.

Parallel with this abnormal collection of cholesterin the quantity of substances which keep it in solution, as fatty acids and biliary salts, is gradually diminished. The diminution of the quantity of fatty acids is due to the insufficiency in the assimilation of fats; the decrease of biliary salts is due to the atony of the liver. Besides the above-mentioned conditions there are two more circumstances, which aid in the precipitation of cholesterin: (1) the acidity of the bile, which is equivalent to the total acidity of all the juices of the system in this dyscrasia; (2) the superabundance of lime in the bile, which is influenced by the superabundant presence of acids in the juices of the system.

All these cases lead to the extraction of cholesterin from solution, according to the easily understood chemical mechanism.

At the Medical Congress in 1891, held in Wiesbaden, Naunyn critically explained with great details, and in a most efficient and noteworthy manner, the dyscratic theory of Bouchard. He brought out against it excellent and important arguments: (1) the constant and unchangeable presence of the cholesterin and lime in bile, without any relation to food digested; (2) the presence of substances in the bile, which aid the solution of cholesterin, in much greater quantity than is necessary to keep the cholesterin in solution.

Naunyn gave great quantities of lime salts, and still could not bring about their increase in the bile. We must mention here also this fact, that the acidity of bile in the dyscrasia has not as yet been determined. Finally, the constant presence of inflammatory or congestive changes, acute or chronic, in the ways and byways through which we know the bile must flow, cannot be explained by this theory. Hence we see on what foundation rests this, at first sight truly attractive, theory of Bouchard.

Until very recent years the knowledge of the causes of cholelithiasis did not differ very much from the views held by authors 100 or 150 years ago. The insufficient and unexplanatory causes of cholelithiasis usually brought forth by authors were most evident to all scientific thinkers. Few, if any, could or would believe that stasis or condensation of bile, due to old age, sedentary or inactive life, slower circulation of blood, difficulties of motion of chest or abdomen, due to tight dressing, corsets or pregnancy (the latter usually being given as the cause of cholelithiasis in women) and many other similar conditions, were the causes of gall-stones. The critical examination of all these causations, leads us to but one undeniable fact—namely, that all which favors the slowing of the flow of bile, favors also the formation of gall stones.

Modern investigations and teachings on the pathogenesis of cholelithiasis, shows us that the disease is but a local one, bringing about as a result the morbid condition of the bile ducts and gall bladder, and that this condition is uninfluenced by any disturbance in nutrition. We have learned that this view is not a new one. Lately, Naunyn became a most ardent defender of this theory. This investigator has mostly influenced that there is now a change in the views on the causation of gall stones.

From a chemical point of view, the entire morbid condition of cholelithiasis is but a precipitation of certain normal constituents of bile; cholesterin, which is held in solution, as we have said before, by the presence of biliary salts and fatty acids and bilirubin, which forms with lime an insoluble compound. Hence it is but necessary to answer the following question: What are the conditions that bring about the precipitation of these normal compounds of the bile from the solution? The vast majority of gall stones are composed of cholesterin and bilirubin united with lime. Only in exceptional cases does cholesterin form the basis of the stone; usually this part is taken by the calcium salt of bilirubin. Calcium bilirubianate is first precipitated, later this kernel or base, is surrounded by cholesterin, either pure, or in combination with the calcium compounds of bilirubin. Sometimes gall stones are composed exclusively of the combination of bilirubin and lime—generally in the intrahepatic ducts. These facts prove that the conditions which bring about gall stones are those which aid in the precipitation and union of bilirubin with lime. Normally, bilirubin is not found in bile in a chemical union with lime. Even the addition of a great quantity of lime does not aid the formation of this union. The presence of albumin, however, brings about the precipitation of bilirubin, if we add to the solution but a few drops of lime water (Naunyn's experiment). Hence the presence of albumin in the biliary ducts, favors the formation of compounds of bilirubianate of calcium. This condition we find in the catarrh of biliary ducts, where the dissolution of membranous tissue provides the necessary albumin.

As far as cholesterin is concerned, it is also a creation of the destruction of cells. We find it in all atheromatic, carcinomatous, tubercular and purulent substances, especially in catarrhal disturbances of the mucous membranes (sputum in bronchitis, pulmonary tuberculosis, and the like). It is the opinion of Naunyn, that cholesterin appears in bile in much the same manner.

From the data mentioned it follows that the conditions in which there is the precipitation of the cholesterin and bilirubin, have a local character and are but the destruction of the cells of membranes, which accompanies the inflammatory complications of the gall bladder and ducts.

As was mentioned above, these various agents which have once been considered as the momentous causes and of great value in the production of cholelithiasis, simply act in the direction that they predispose to the stasis of bile in the gall bladder. But this stasis in itself is not able to bring about the inflammation of the mucous membranes of the biliary ducts. This latter is effected only by the fact that the stagnation of bile wonderfully favors the inflammation of biliary ducts and gall bladder. Hence, the inflammation of the mucous membranes of the bladder and bile ducts and the resulting catarrh of the membrane are the most necessary *sine qua non* conditions of the cholelithiatic process (Naunyn).

The bacterial theory, to which I now turn my attention, is, as we see, but the fulfillment of the local theory of causation of cholelithiasis. It is, we may say, as old as bacteriology itself. From its very origin of existence until most recent years, the parasitic theory of cholelithiasis met with great distrust, equally from the great medical circles as from the learned scientific investigators. The results, however, which have been attained in the last few years, are principally the results of investigations by Gilbert and Mignot (1898), and these furnish the parasitic theory with most solid scientific foundations.

When in 1886, Galippe proved the presence of microörganisms in the salivary calculi, and announced the opinion that all calculi which are met in the animal system are the result of chemical action, produced by microörganisms on the fluids of the system, he found but few believers, especially in its relation to the pathogenesis of cholelithiasis. The physicians and pathologists had an unwavering faith in the antiseptic properties of bile. It appeared at that time as an impossibility, that microörganisms could reach the normal bile, thrive, grow and multiply there. In a short time, however, Naunyn proved that the last part of ductus choledochus is usually not free from microörganisms, and later it was shown (by Vignal, Copeman, Winston, and Bernabel) that bile does not possess antiseptic properties.

In 1891, Létienne comes to the conclusion, after long and most thorough bacteriological investigations, that the presence of microörganisms in the biliary ducts always brings about the precipitation of various compounds of bile. But what is the nature of these microörganisms which are found in the biliary calculi? How often are they found there? What is the cause that one calculus may have within itself microörganisms, while another does not contain them? Whether the bacteria found in calculi are alive and capable of growth or dead? Why are they alive in one while dead in another? When do the bacteria penetrate into the calculi? These and other questions were propounded, which at that time could not be given an answer. In the meantime from the answers given to these questions depended the solution of the fundamental question: Is cholelithiasis bacteriological in origin? Gilbert began to study and tried to elucidate this side of the pathology of bile.

At first he was assisted by Dominici, (1894), later by Fournier, (1896.) These investigators proved that bacillus coli was usually the microörganism of biliary calculi; that is, the same microörganism which most frequently produces the inflammation of gall bladder and biliary ducts. But soon investigations proved that also other pathogenic microörganisms are found in gall stones, as bacillus Eberthi, staphylococcus, streptococcus and others. Bacillus Escherichi, that is bacillus coli, was found either dead or alive in one third of the total number of cases investigated—seventy. Then was also established the fact that calculi which were inhabited by live microorganisms were late formations, while old calculi were sterile, or only contains remnants of dead microörganisms.

Finally, on the most thorough and special researches, the above mentioned investigators proved that among calculi which were but lately formed, some were penetrable to the microörganisms while others were not, and hence it follows that microörganisms existed in the bile even before the formation of calculi.

These new spoils in the field of bacteriology of cholelithiasis soon earned wide reputation; in a short time, however, it was noticed that their substantiative value was much over-rated. And truly so; for all adherents of the dyscratic theory agree that the presence of calculi in biliary ducts most excellently favors their infection. When the biliary ducts become infected the calculus itself may be infected; the least fissure, the least injury of the external layers may be the cause that microörganisms which have emigrated from the intestines may soon find themselves in the calculus. This impenetration of the microörganisms may occur even in unmarred calculi, as *e. g.*, in cholesterin calculi, not covered by any bilirubinate of calcium layer; it may also be proved experimentally (Gilbert et Fournier).

Admitting that all calculi are thoroughly unpenetrable by the microörganisms, what would the presence of these organisms prove when found in the gall stones? Only this, that the microörganisms existed in the bile at the time of the formation of the stones, and surely not that they brought about their very existence. But this is not all. The frequent sterility of the gall stones—forty-three times out of seventy—provides the adversaries of the parasitic theory one excellent argument. The fact that experiments tending to produce cholelithiasis have failed (Fournier, 1896), it also seems to argue against this theory. Hence the bacteriological theory of cholelithiasis did not make great progress in 1896, as compared with the works of Naunyn and Létienne of 1891.

Finally, in the beginning of 1897, Gilbert, assisted by Dominici and Fournier, found in the gall bladder of a dog infected with bacillus coli, a well-formed calculus. The same discovery was made in a gall bladder of a guinea-pig and a rabbit, in the same year. Gilbert and Fournier while experimenting attained identical results of cholelithiasis, by the infection of the gall bladder with Eberth's bacillus.

Experimental cholelithiasis is of great value to bacteriological theory. We must admit that all the results of experiments at the hands of Gilbert and his students were purely accidental. Mignot, however, brought this experimental technique to such a standard, that what formerly was the result of a happy coincidence of circumstances, now became the regular and constant rule.

Mignot, in 1896, in a doctorate thesis, has shown that foreign bodies when sterile can remain in gall bladder for a long time and not produce inflammation or any sediment in the bile. The formation of calculi does not occur even when there is a stagnation with a partial renovation of bile in the gall bladder by the presence of a foreign aseptic body. Notwithstanding the large size of the foreign substance, and its presence for a great length of time in the bladder the walls of the later remain translucent and do not show either any thickening or inflammation and the bile does not become turbid. According to Mignot neither does the action on the walls of the bladder of a foreign substance, infected by septic microörganisms aid the formation of calculi, or the sediment which is adhering to foreign substances. As an expression of tendency toward the formation of calculi, we can mention the formation of biliary sediment mixed with pus. In all cases where virulent microörganisms were used for infection the cholecystitis was very intense, in fact more intense than is usual in cases of cholecystitis calculosa without any special complications.

In experimental productions of cholelithiasis the most important of all is the question of microörganisms. The kind of microörganism seems to play only a secondary rôle. It is necessary that the given bacteria thrive in the bile and decompose the same, and this is the common property of many kinds of microörganisms. From the great number of these great microörganisms I can mention: bacillus coli, bacillus typhi abdominalis, staphylococcus, streptococcus, even bacillus subtilis, not a pathogenic microörganism, brings about cholelithiasis in animals. The theory of two kinds of cholelithiasis, due to bacillus coli and bacillus typhi abdominalis (Gilbert and Fournier) means only that these two microörganisms are most commonly met with in human cholelithiasis and does not answer the universally known facts. The virulence of microörganisms is more important than the kind of microörganisms. It must be as mild as possible and yet at the same time must retain the faculty of vegetation and multiplication in the bile. After many attempts at rendering the microörganisms as little virulent as possible. Mignot came to this most simple and certain method. The given bacteria are cultured for several months in bile, to which is gradually added a lesser quantity of bouillon, or the serous fluid from abdominal dropsy. In this way we may obtain pure bile; but then, it often happens that the culture dies. In the bile, with a little addition of bouillon, we can for a long time keep the culture, which is most excellent for the formation of biliary calculi. Microörganisms prepared in such a manner should not produce pus when injected hypodermatically into the animal.

But the injection of a weakened culture into the gall bladder of an animal will not suffice for the production of cholelithiasis, for a parasite which is so slightly virulent would not be able to grow on the mucous membrane; when swept away by the bile sooner or later it would be expelled from the intestine and the sediment formed in the bile would not find in the healthy mucous membrane any point around which it might gather and amass.

The microörganism must be given sufficient time to implant itself well in the mucous membrane, hence for the purpose it is well to place in the gall bladder a foreign substance, fairly large and porous, which should act as a shelter for the multiplying microörganisms—a cotton tampon or piece of fibrous material. After ten or fifteen days the tampon is removed. If a small foreign body be later introduced into the gall bladder, and the substance be attached to the wall, in order to prevent its expulsion from the bladder, we can be sure that in one or more months, we shall find around the foreign body a calculus composed of cholesterin and biliary coloring matter, the calculus being the larger, the longer the time elapsed from the operation. The smaller the foreign substance, the better the formation. In case a tampon be infected by a given nonvirulent culture and remain in the bladder for three weeks, we can in a few months after the extraction of the tampon, without permanently introducing a foreign body, also obtain biliary calculi, but the results will not be as lasting. In such proceedings Mignot obtained in more than one third cases true biliary stones stratified and almost exclusively composed of cholesterin.

From his experiments Mignot concludes that cholelithiasis is easily produced when the biliary ducts of the animal are in certain described circumstances, the most important of these being: (1) A slight catarrh of the mucous membrane, due to the growth of microörganisms almost void of any virulency; (2) A conditional atony of the gall bladder, or generally, a complete stasis. In complete stasis the formation of true stratified calculi is impossible.

As long as bile can renew itself around the forming stones and as long as there is a bacterial fermentation of the bile, so long the calculi can increase in size. It is impossible to admit that there is an increase of the calculi after the death of the microörganisms present (in man their death occurs in a short time); in fact, supposing that precipitation of bile is due directly to the action of the microörganisms or secondary to the results of the catarrh of the mucous membrane, the death of the microörganisms in both cases will stop the formation of sediment. Invariably, if we remove from the gall bladder the calculi in the period of their growth, with great probability new stones will form there. Hence the so-called cholecystomia idealis, that is the removal of the calculi and direct closure of the bladder, is only called for or directed, when a former examination will convince of the aseptic condition of the bile. Calculi obtained in an experimental way are equally, both as to their physical and chemical appearance, entirely similar to biliary calculi as seen in man. There is a complete analogy also from the bacteriological point of view. Still there occurs a difference, which, however, often a close inspection, shows itself to be but a simulating one.

In almost two-thirds of the cases of cholelithiasis in man, the bile is sterile. This circumstance is easily explained. Bacteria cultured in pure bile die very easily. The same can occur in the biliary ducts of man. It is true that the author has never seen anything like it in his experiments, but then, the time of length of experiment cannot be prepared with the great length of time of human cholelithiasis. And even Mignot says that in one case the bile was entirely sterile, while in the center of the calculus microörganisms have been found. We must add that in animals cholelithiasis was most easily provoked, if for the purpose of experimentation, were used microörganisms taken from cholocystitis calculosa of man.

The anatomo-pathological conditions have still greater similarity. All the authors who have studied the anatomical changes of the biliary ducts in cholelithiasis, have always noticed an inflammation in new, and hardening and disappearance in old cases. Mignot in his experiments on animals has found almost all the anatomo-pathological changes, which have been described in man. The results obtained by Mignot, Gilbert, Dominici and Fournier, are without question of greatest importance to pathology. The value of these experimental facts, however, cannot be as yet fully substantiated.

Though the very fact of possibility of experimental cholelithiasis is at present without any doubt, still there must be controlling and complete experiment, which in view of recent events, especially of Mignot's investigations, have as yet not been made. Moreover the fact that by the infection of the mucous membrane of the gall bladder we can bring about cholelithiasis, does not prove that the pathogenesis of this disease in man is always the same (Gilbert); for, though cirrhosis of the liver —using Gilbert's comparison—can be produced by the infection of tubercular bacilli in guinea-pigs, can we come to the conclusion that cirrhosis of the liver in man is also of parasitic origin? Similarly, that in an animal, under the influence of lead poisoning there is very early cirrhosis of the kidneys, are we, therefore, authorised to come to the conclusion that interstitial nephritis in man can also result from the same poisoning?

There is no doubt that the production of cholelithiasis in an experimental way forms quite a strong foundation for the bacterial theory; bacteriological investigations, which have been made in the cholelithiasis of man, find in this very fact a most important completion. But, perhaps in the matter of the formation of biliary calculi, it is not the microörganisms that play the important rôle, but rather the catarrh of the biliary ducts which they produce. In such a case, besides the microörganisms, there may be other factors which can provoke cholelithiasis. Such reasoning, in fact, has forced certain authors, to build a new hypothesis, or rather a slight change from the theory of local production of cholelithiasis. According to this theory, cholelithiasis is due purely to local changes—namely, to the catarrh of biliary ducts. In the majority of cases the catarrh is the result of infection, or perhaps in view of this theory, it may be from some other cause. Certain formations are expelled from the system by means of the bile. Their passage through the biliary ducts may produce the congestion or inflammation of the latter. Hunter, a follower of this theory, has really shown that in a dog, the injection of toluytendyamin produced intense jaundice, with thickening and stickiness of the bile. This condition of the bile, according to Hunter, depends on the catarrh of all the biliary ducts, produced by the passage of poison or its derivatives.

According to this author in chronic dyspepsia, which is so common in people of sedentary habits, there are conditions for the production of similar toxins and their passage through the biliary ducts. Such, according to Hunter, is the beginning of small formations, which are found even in the last branches of the biliary ducts, from there, they later come with the flow of the bile to the bladder and are the kernels of large calculi. Cholelithiasis of toxic origin is but an hypothesis, which as yet has no foundation on a convincing fact.

BIBLIOGRAPHY.

1. R. Mignot. L'origine microbienne des calculs biliaires. Archives Gènér de Medecine, 1898, August and September.

2. A. Gilbert. Note pour servir á l'histoire de la theorie microbienne de le lithiase biliaire. Arch. Gènér de Med., 1898, September 3.

3. W. Hunter. Causes de la lithiase biliaire. British Medical Journal, October 30, 1897; according to the report of the meeting of the British Medical Association in Arch. Gènér de Med., 1898, August.

4. Gilbert et Fournier. Pathogénie de la lithiase biliaire. Presse Med., 1898, No. 41.

5. S. Pechkranc. Streszczenia zbiorowe. Patogeneza Kamicy zólciowej w swietle najnowszych badan. Medycyna. Tom XXVII., Nos. 6 and 7.

508 FILLMORE AVENUE.

